# Regulation of Histone Emulsification by HPDL via LDHA/LDHB Promotes EC Cell Proliferation

**DOI:** 10.32604/or.2026.068833

**Published:** 2026-04-22

**Authors:** Yan Wang, Jialei Zhu, Shiyang Wei, Lijie Jin, Zhanqiu Liu, Jie Xu, Nana Yang, Xuefeng Jiang, Caizhi Wang, Lingling Wang

**Affiliations:** 1 Department of Obstetrics and Gynecology, The First Affiliated Hospital of Bengbu Medical College, Bengbu, China; 2 Department of Gynecology, Huaiyuan County Hospital of Traditional Chinese Medicine, Bengbu, China; 3 Department of Gynecology, People’s Hospital of Guangxi Zhuang Autonomous Region, Qingxiu District, Nanning, China; 4 Department of Gynecology, The First Affiliated Hospital of Jinan University, Guangzhou, China

**Keywords:** Endometrial cancer, histone lactylation, 4-hydroxyphenylpyruvate dioxygenase-like protein, lactate dehydrogenase A/B, cell proliferation

## Abstract

**Background:**

The role of 4-hydroxyphenylpyruvate dioxygenase-like protein (HPDL) in endometrial cancer (EC) progression remains poorly understood, particularly its involvement in metabolic-epigenetic crosstalk via lactate-driven histone lactylation. This study aimed to investigate HPDL’s mechanistic contribution to EC pathogenesis.

**Methods:**

Stable HPDL-overexpressing and knockdown EC cell lines (HEC-1-B and AN3CA) were generated using lentiviral vectors. Functional assays (proliferation, migration, invasion), subcutaneous xenograft models in BALB/c nude mice, and molecular analyses were conducted. Lactate levels, Pan-lysine lactylation (pan-kla), histone H3K18 lactylation (H3K18la), and effects of sodium oxamate (lactate modulator) were assessed. Lactate Dehydrogenase A/Lactate Dehydrogenase B (LDHA/LDHB) knockdown, promoter activity assays, and chromatin immunoprecipitation (ChIP) were performed to evaluate H3K18la occupancy at LDHA/LDHB promoters.

**Results:**

HPDL knockdown reduced intracellular lactate, Pan-Kla, and H3K18la levels, while overexpression elevated these markers. Sodium oxamate amplified lactate and lactylation in HPDL-overexpressing cells but suppressed histone lactylation independently of HPDL. LDHA/LDHB knockdown diminished lactylation, repressed HPDL expression, and inhibited promoter activity. ChIP revealed H3K18la enrichment at LDHA/LDHB promoters in HPDL-overexpressing cells and reduced occupancy in knockdown models. HPDL enhanced EC cell proliferation, migration, and invasion *in vitro*. *In vivo*, HPDL-overexpressing xenografts exhibited accelerated tumor growth and larger volumes compared to controls.

**Conclusions:**

HPDL regulates histone lactylation via LDHA/LDHB and promotes the proliferation of EC cells.

## 1 Introduction

Endometrial cancer (EC), the third leading cause of gynecological cancer-related deaths in women [1]. The poor prognosis of advanced EC is primarily driven by late diagnosis and acquired therapy resistance, with 5-year survival dropping from 95% in Stage I to 17% in Stage IV [2]. In 2022 alone, the American Cancer Society reported approximately 66,000 new EC cases and 13,000 deaths in the United States, underscoring its growing clinical burden [3]. Emerging evidence highlights lactate as a dual-function metabolite in tumor biology, serving not only as a key carbon source for cellular metabolism but also as a signaling molecule within the tumor microenvironment (TME). Under hypoxic or semi-hypoxic TME conditions, tumor cells secrete excess lactate, fostering a protumoral niche linked to metastasis and poor clinical outcomes [4,5]. Notably, lactate also acts as a substrate for histone lactylation—a novel posttranslational modification implicated in epigenetic regulation [6].

In our prior work, we leveraged The Cancer Genome Atlas (TCGA) database to construct a lactate-related prognostic model for EC, identifying 4-hydroxyphenylpyruvate dioxygenase-like (HPDL) as a key candidate gene. HPDL is an intronless protein-coding gene encoding a mitochondrial enzyme with putative 4-hydroxyphenylpyruvate dioxygenase activity, as annotated in Gene Ontology (GO) databases [7,8]. While HPDL mutations are associated with autosomal recessive neurodevelopmental disorders [9], recent studies reveal their oncogenic role in metabolic reprogramming. For instance, HPDL promotes glutamine metabolism to mitigate oxidative stress in pancreatic ductal adenocarcinoma [10].

To elucidate HPDL’s mechanism in EC, we performed GO/Kyoto Encyclopedia of Genes and Genomes (KEGG) pathway analyses on HPDL and its positively correlated genes (correlation coefficient >0.3). These genes were enriched in pathways such as fatty acid metabolism, mitochondrial metabolism, and central carbon metabolism in cancer, a pathway directly linked to lactate dehydrogenase A (LDHA) and B (LDHB). Both LDHA and LDHB, established oncogenes, are overexpressed in EC and exhibit a positive correlation with HPDL expression. We hypothesize that HPDL drives EC progression by upregulating LDHA/LDHB, potentially through histone lactylation-mediated epigenetic regulation.

This study aims to investigate whether HPDL enhances EC cell proliferation and worsens patient prognosis by upregulating LDHA/LDHB via histone lactylation. Through cellular and animal-level experiments, we seek to validate this hypothesis, thereby clarifying the roles of HPDL and histone lactylation in EC pathogenesis. Our findings may provide a foundation for novel therapeutic strategies targeting lactate metabolism in endometrial cancer.

## 2 Materials and Methods

### 2.1 Ethics Approval and Compliance Statement

This study was approved by the Ethics Committee of the First Affiliated Hospital of Bengbu Medical College (Approval No. 2023432). The experimental animal protocol was approved by the Institutional Animal Care and Use Committee (IACUC) of Jinan University (ethics approval number: IACUC-20230106-01). All methods were carried out in accordance with relevant guidelines and regulations, including the Declaration of Helsinki for human studies, ARRIVE guidelines for animal research, etc. Written informed consent was obtained from all participants. A total of 160 tissue samples were collected, including 80 EC tissue samples from patients diagnosed with EC and 80 normal endometrial tissue samples from control patients. All samples were obtained from individuals who underwent surgical procedures at the First Affiliated Hospital of Bengbu Medical College between January 2021 and December 2022.

### 2.2 Cell Line Acquisition and Culture

HEC-1-B and AN3CA EC cell lines were authenticated by STR profiling (American Type Culture Collection (ATCC), Manassas, VA, USA, Cat. No. CL-0100) and consistently tested negative for mycoplasma contamination using the MycoFluor™ Mycoplasma Detection Kit (Thermo Fisher, Waltham, MA, USA, Cat. No. M-7006) in monthly screenings. Cells were cultured in Minimum Essential Medium (MEM; Wuhan Procell Life Technology Co., Ltd., Cat. No. PM150410) under standardized conditions: a humidified atmosphere containing 5% CO_2_ at 37°C. The culture medium was replaced every 24–48 h, and cell growth status was regularly monitored. Subculturing was performed when cells reached 90% confluence to maintain optimal proliferation.

### 2.3 Immunofluorescence Analysis

After 48 h of lentiviral infection (for knockdown) or plasmid transfection, three distinct plasmids (SiHPDL1178181, SiHPDL1178182, SiHPDL1178183 (GeneChem, Shanghai, China)) were used to transiently knock down HPDL expression in EC cell lines (AN3CA and HEC-1-B cells). Forty-eight hours post-transfection, the transfection efficiency of different plasmids was compared by measuring fluorescence intensity. Results indicated that SiHPDL1178181 exhibited the highest transfection efficiency. This plasmid was packaged into lentivirus (SiHPDL1178181, Jike Gene) and lentivirus (LV) (LV-HPDL (28880-1; GeneChem)). AN3CA and HEC-1-B cells were washed three times with 1× PBS (pH 7.4), detached with 0.25% trypsin, and seeded into 2 cm diameter confocal imaging dishes at a density of 1 × 10^5^ cells per dish. Following a 12–24 h incubation period to ensure complete adherence, cells were removed from the incubator and subjected to three washes with room temperature (RT) PBS. Subsequent steps included fixation, permeabilization, and blocking, followed by overnight incubation at 4°C with shaking in primary antibody solutions. Antibody dilutions were as follows: anti-H3K18lac (PTM BioLab, Inc, Hangzhou, China, Cat. No. 1406, dilution 1:50), anti-pan kla (PTMBIO, Hangzhou, China; Cat. No. 1401, dilution 1:50), anti-HPDL (Proteintech, Wuhan, China, Cat. No. 20777-1-AP, dilution 1:20), anti-H3 (Abbkine Scientific Co. Ltd., Wuhan, China, Cat. No. A01070, dilution 1:100), and anti-LDHA/LDHB (Proteintech, Cat. No. 19987-1-AP, dilution 1:50). Cells were then incubated with Goat Anti-Rabbit IgG H&L/FITC antibody (BIOS Technology Co., Ltd., Taichung, China, Cat. No. bs-0295G-FITC, dilution 1:200) for 2 h at RT in the dark. After completing the immunostaining protocol, samples were imaged using a confocal microscope (Leica Microsystems, Ernst-Leitz-Str. 17-37, 35578 Wetzlar, Germany, STELLARIS 5/STELLARIS 8).

### 2.4 Scratch Assay Protocol

A 6-well plate was pre-marked on the underside with three parallel horizontal lines spaced 1 cm apart, spanning the diameter of each well. AN3CA and HEC-1-B Cells (5 × 10^5^ per well) were seeded and incubated for 24 h to achieve >90% confluency. At 24 h post-seeding, the plate was removed from the incubator, and uniform longitudinal scratches were created perpendicular to the pre-drawn horizontal lines using a vertically held 1 mL pipette tip. Detached cells were removed by washing three times with 1× PBS (pH 7.4) followed by the addition of fresh serum- and antibiotic-free MEM medium. Initial scratch widths were documented via imaging (timepoint = 0 h). Post-treatment, the plate was re-incubated, with medium refreshed at designated intervals. Migration progress was monitored by imaging scratches at 6, 12, 24, and 48 h. Scratch closure was quantified using ImageJ software (National Institutes of Health (NIH), Bethesda, MD, USA, Version: 1.53t-1) to measure intercellular distances at each time point.

### 2.5 Plate Colony Formation Assay

The AN3CA and HEC-1-B HPDL overexpression cell lines, along with their respective knockdown cell lines and parental EC cell lines, all exhibiting good growth status, were detached and counted after being washed three times with 1× PBS (pH 7.4). Each cell line was seeded in 6-well plates at a density of either 500 or 1000 cells per well. The medium was replaced every 3–4 days, and colony formation was monitored. After approximately 10–14 days, colony formation was visible under the microscope (Leica Microsystems, STELLARIS 5/STELLARIS 8). Subsequently, the cells were washed three times with PBS, and 1 mL of 4% paraformaldehyde was added to each of the six wells for fixation, which lasted for 30 min. The cells were then washed three times with PBS, and 1 mL of methanol was added to each of the six wells for permeabilization, which took 20 min. Finally, the cells were washed three times with PBS, 1 mL of a 0.1% crystal violet solution was added to each of the six wells for staining, which lasted for 30 min, and after being washed three times with PBS, the cells were dried and photographed.

### 2.6 Cell Viability Assay (CCK-8 Assay)

The AN3CA and HEC-1-B HPDL overexpression cell lines, along with the knockdown cell lines and the parental EC cell lines, all exhibiting good growth status, were washed three times with 1× PBS (pH 7.4), detached, counted, and seeded into 96-well plates at a density of 500 cells per well (with 5 replicate wells per group). After the cells had attached to the plates, the culture was continued for 24, 48, 72, 96, and 120 h. At each of these time points, the cells were transferred to new 96-well plates, 100 μl of medium and 10 μl of Cell Counting Kit-8 (CCK-8) (Beyotime, Shanghai, China, Cat. No. C0038) were added to each well, and the plates were incubated for 0.5 to 2 h. The optical density (OD) was measured at 450 nm using a microplate reader (Nikon Eclipse, Tokyo, Japan, TE 300) during this incubation period.

### 2.7 Transwell Migration Assay

AN3CA and HEC-1-B cells (including HPDL-overexpressing, knockdown, and parental EC cell lines) in the logarithmic growth phase were washed three times with 1× PBS (pH 7.4), detached, and counted. A 200 μl suspension of serum-free MEM containing 1 × 10^5^ cells was added to the upper chamber of the Transwell apparatus (Thermo Fisher, Cat. No. 141002) while the lower chamber was filled with 500 μl of medium containing 20% fetal bovine serum (FBS; Biosharp, Hefei, China, Cat. No. BL302A). After 24 h of incubation, cells were washed three times with PBS, fixed with 4% paraformaldehyde (30 min), permeabilized with methanol (Chemical Book, 67-56-1) (20 min), and stained with 0.1% crystal violet (30 min at room temperature). Following two additional PBS washes and air-drying, non-migrated cells on the upper chamber surface were gently removed using cotton swabs. Migrated cells on the lower membrane surface were quantified under a fluorescence microscope (Nikon Elipse TE 300, Tokyo, Japan). We added the histone lactylation inhibitor sodium oxamate (Absin, Shanghai, China, CAS 811929) to the HPDL overexpression group and observed the change in cell invasion in the HPDL overexpression group after lactate inhibition. We added the histone lactation enhancer Sodium L-lactate (L-NaLa; Sigma, Taufkirchen, Germany, Cat. No. 71718) to the HPDL knockdown group to observe the alteration of cell invasion in the HPDL knockdown group after histone lactate inhibition.

### 2.8 Flow Cytometric Analysis of the Cell Cycle, Histone

Logarithmic-phase AN3CA and HEC-1-B HPDL-overexpressing cells, knockdown cells, and parental EC cell lines were washed three times with 1× PBS (pH 7.4) and detached for counting. The cells were then seeded in 6-well plates (2 × 10^5^ cells per well), and the plates were incubated for 24 h. One milliliter of the prepared cell suspension was centrifuged, and the supernatant was removed. Next, 500 μl of 70% cold ethanol was added, and the cells were fixed at 4°C for 2 h to overnight. After washing the cells with PBS to remove the fixative, 100 μl of RNase A was added, and the cells were incubated in a 37°C water bath for 30 min. Then, 400 μl of BeyoFC™ PI Staining Solution (Beyotime, Cat. No. C1734) was added to each well. After staining for 15 min in the dark, the cells were injected into a flow cytometer (BD Biosciences, BD FACSymphony™, Milpitas, CA, USA) for detection, and red fluorescence at an excitation wavelength of 488 nm was recorded.

### 2.9 Chromatin Immunoprecipitation (ChIP)-Quantitative Polymerase Chain Reaction (qPCR) Assay

AN3CA and HEC-1-B HPDL overexpression cell lines, along with their corresponding knockdown cell lines and parental EC cell lines, were cultured in 10 cm dishes containing 10 mL of complete medium. Once they reached over 90% confluence, the cells were harvested and stored. For crosslinking, 270 μl of 37% formaldehyde was added directly to the culture medium and gently mixed to achieve a final formaldehyde concentration of 1%. Chromatin immunoprecipitation (ChIP) was performed using a commercial ChIP Assay Kit (Beyotime, Shanghai, China, Cat. No. P2078) according to the manufacturer’s instructions. The immunoprecipitated chromatin was then purified and subjected to qPCR analysis. RNA reverse transcription: RNA sample mixed and centrifuged, and placed on ice for later use. The TAKARA (Suzhou Novoprotein Scientific Inc, Suzhou, China, Cat. No. E096) reagent kit is melted on ice, shaken first, and then centrifuged. Reverse transcription system 20 μl system: Reagent 14 μl + (2, 3, 4) each 1 μl + 5 (the remaining liquid is replenished to 20 μl) + total RNA sample 1 μg. Mix and centrifuge after adding the sample. Place it on the reverse transcriptase, adjust the parameters: 37°C, 15 min; 85°C, 5 s; ends at 4°C. The cDNA obtained by reversing the qPCR amplification step is used as the sample. The reaction system is configured with 10 μl of system per well, and 3 sub-wells are set for each sample. The total amount of the 3 wells is prepared together. After adding the sample, mix well, exhaust and put it into an 8-tube, and wait for detection in the dark. PCR amplification instrument (Bio-Rad, Shanghai, China, Cat. No. S1000) setting parameters: preheat at 95°C for 20 s; 40 cycles: 15 s at 95°C, 60 s at 60°C. Record the cycle threshold (Ct) of each gene in the experiment and use the 2^−ΔΔCt^ method to calculate the mRNA expression of the target gene. The PCR primers are shown in Table A2.

### 2.10 Western Blotting (WB)

Nuclear proteins were extracted from 50 mg of endometrial tissue or cultured cells using a nuclear and cytoplasmic protein extraction kit. The extracted nuclear proteins were aliquoted and stored at –80°C for subsequent use. The Western blotting procedure included gel preparation, electrophoresis, membrane transfer, membrane washing, and antibody incubation. Primary antibodies were diluted as follows: anti-H3K18lac (PTMBIO, Cat. No. 1406, Dilution 1:2000), anti-pantothenic acid anti-pan Kla (PTMBIO, Cat. No. 1401, Dilution 1:1000) (1:50 is redundant here and should be removed), anti-HPDL (Proteintech, Cat. No. 20777-1-AP, Dilution 1:1000), anti-H3 (Abbkine, Cat. No. A01070, Dilution 1:3000), and anti-LDHA/LDHB (Proteintech, Cat. No. 19987-1-AP, Dilution 1:2000). Membranes were incubated with the primary antibodies at 4°C overnight. Subsequently, membranes were incubated with a horseradish peroxidase (HRP)-conjugated goat anti-rabbit (Abbkine, Cat. No. BMS005, Dilution 1:7000) for 2 h at room temperature. After further washing, the blots were subjected to chemiluminescent detection. The histone lactylation inhibitor sodium oxamate (MedChemExpress, Monmouth Junction, NJ, USA, CAS No. 565-73-1) was added to HPDL-overexpressing HEC-1-B cells at concentrations of 0, 10, 20, and 30 mmol. WB analysis showed that the signals of the anti-Pan Kla and anti-H3K18lac antibodies were significantly reduced in these cells. Antibodies & Detection Reagents in Table A1.

### 2.11 Plasmid Transfection and Lentiviral Transfection

In cell culture, when cells grow to approximately 90% confluence, they are seeded into 6-well plates (e.g., for AN3CA cells: T25 flasks are seeded the previous morning, and after 24 h, the cell density reaches 80%–90%, ready for transfection. HEC-1-B cells grow slightly slower, typically reaching over 80% confluence after 48 h. HPDL knockdown plasmids (with catalog numbers SiHPDL11781181, SiHPDL11781182, and SiHPDL11781183) and an overexpression plasmid, both carrying green fluorescent protein (LV-HPDL (28880-1), are added. Transfection is carried out for 48–72 h, after which RNA and protein are harvested in advance for subsequent experiments. For lentiviral transfection, the viral solution is diluted. One aliquot of lentivirus and transfection reagent A (HitransG A, Cat. No. REV005) is taken out. The viral titer for the HPDL knockdown strain is 1 × 10^9^, and for the HPDL overexpression virus, it is 3 × 10^9^. The viral solution is prepared at a 1:50 ratio: For the HPDL knockdown strain: 4 μl of virus + 196 μl of MEM medium. For the HPDL overexpression virus: 2 μl of virus + 198 μl of MEM medium. For the control (con): 3 μl of virus + 197 μl of MEM medium.

### 2.12 Laboratory Animals

Forty female BALB/c Anu nude mice (4 weeks old; 14–16 g; Beijing Huafu Kang Co., Ltd., strain code 490; RRID: IMSR_APC:001) were housed under specific pathogen-free (SPF) barrier conditions. Randomly divided into 4 groups, with 5 mice in each group, representing variants of the AN3CA cell line—including HPDL overexpression group, HPDL knockout group, siNC transfection group, and parental AN3CA group. Cells from each group were amplified in 10-cm culture dishes. Cells were harvested at >90% confluence during logarithmic growth phase, following confirmation of mycoplasma—and bacterial—free status. Subsequently, 5 × 10^6^ cells of each variant were subcutaneously inoculated into each mouse. Tumor dimensions were measured blindly by two independent investigators using digital calipers, with volume calculated as (V = π/6 × L × W^2^), L represents Length (the longest dimension of the tumor), while W represents Width (the shorter dimension perpendicular to the length). Humane endpoints were strictly enforced: tumor volume >1500 mm^3^ or >20% body weight loss. All animal procedures were approved by the Jinan University Institutional Animal Care and Use Committee (IACUC Approval #: IACUC-20230106-01) in accordance with institutional guidelines.

### 2.13 Data Analysis Methods

The Cancer Genome Atlas (TCGA; https://portal.gdc.cancer.gov/, V23.0), Genotype-Tissue Expression project (GTEx) database (https://www.genome.gov/Funded-Programs-Projects/Genotype-Tissue-Expression-Project). GO/KEGG enrichment analyses: Gene Ontology (GO) and KEGG pathway enrichment analyses were performed on differentially expressed genes (DEGs) using the ‘clusterProfiler’ R package (version 4.16.0), R Studio (version 4.2.0) with the top five enriched pathways presented in this study. Gene Set Enrichment Analysis (GSEA) analysis was conducted using ‘ggplot2’ (version 3.3.3), filtering results with a False Discovery Rate (FDR) < 0.25 and *p*.adjust < 0.05.

### 2.14 Statistical Analysis

Data from western blot (WB), cellular immunofluorescence staining, and functional assays—including scratch wound healing, plate colony formation, and Transwell migration/invasion experiments—were analyzed using ImageJ2 Core framework for multidimensional imaging (Bethesda, MD, USA, https://imagej.net/) and GraphPad Prism Version 10.5 (San Diego, CA, USA, https://www.graphpad.com/). Non-parametric statistical tests (Wilcoxon rank-sum test for two-group comparisons or Kruskal-Wallis test for multi-group comparisons) were applied where appropriate. Parametric *t*-tests or chi-square tests were used for pairwise comparisons of normally distributed or categorical data, respectively. A *p*-value < 0.05 was deemed statistically significant.

## 3 Results

### 3.1 The Level of Histone Lactylation Is Elevated in EC Tissues

**Figure 1:**
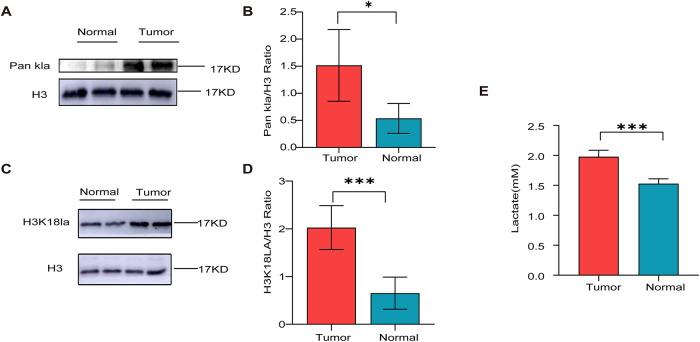

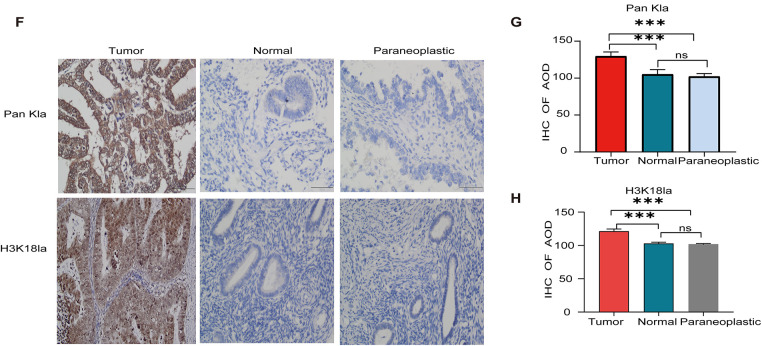
Histone lactylation modification levels in EC tissues. (**A**) Expression of Pan Kla and H3K18la in EC tissues; (**B**) Western blot (WB) quantification results of Pan Kla expression in EC versus normal endometrial tissues; (**C**) WB quantification results of H3K18la expression in EC versus normal endometrial tissues; (**D**) Detection of lactate levels in EC tissues; (**E**) Pan Kla expression in cancer tissues, paracancerous tissues, and normal endometrial tissues with immunohistochemical quantification results; (**F**) H3K18la expression in cancer tissues, paracancerous tissues, and normal endometrial tissues with immunohistochemical quantification results. Lactic acid levels in EC tissues were higher than those in normal endometrial tissues. Scale bar is 100 μm. (**G**) Expression of Pan Kla in EC tissues, normal endometrial tissues, and paracancerous tissues. (**H**) Expression of H3K18la in EC tissues, normal endometrial tissues and paracancerous tissues. *denotes *p* < 0.05, and ***denotes *p* < 0.001. ns denotes no statistical significance

We detected significantly higher levels of pan-histone lactylation (Pan Kla) in endometrial carcinoma tissues compared to normal endometrial tissues via Western blotting (WB) (*p* < 0.05, Fig. 1A,B). Simultaneously, The results of WB showed that the Pan Kla bands in the two EC groups near histone H3, with a molecular weight of 17 kDa, changed significantly. Thus, we hypothesized that lactylation of histone H3 (with a molecular weight of approximately 17 kDa for the modified protein) might be the main factor contributing to the increase in the overall lactylation level in EC patients. Further validation assays revealed that the level of H3K18 lactylation (H3K18la) was significantly increased in EC tissues compared with normal tissues (*p* < 0.001, Fig. 1C,D), consistent with the increased Pan Kla level in EC patients. lactate concentrations were measured in both endometrial carcinoma and normal endometrial tissues, revealing markedly higher lactate levels in the cancerous tissues (*p* < 0.001, Fig. 1E). We used immunohistochemistry to detect Pan Kla and H3K18la in EC tissues and normal endometrial tissues, and the results revealed Pan Kla and H3K18la staining in the nucleus and cytoplasm of EC tissues; Pan Kla was localized predominantly in the cytoplasm, H3K18la was localized predominantly in the nucleus, and the difference was significant (*p* < 0.001). The H3K18la and Pan Kla levels in EC tissues were also significantly higher than those in paracancerous tissues (*p* < 0.001) (Fig. 1F–H). The above results further confirmed that the level of histone lactylation was elevated in EC tissues. The list of clinical cases of EC is provided in (Table A3).

### 3.2 The Expression of HPDL Was Significantly Increased in EC Tissues

**Figure 2:**
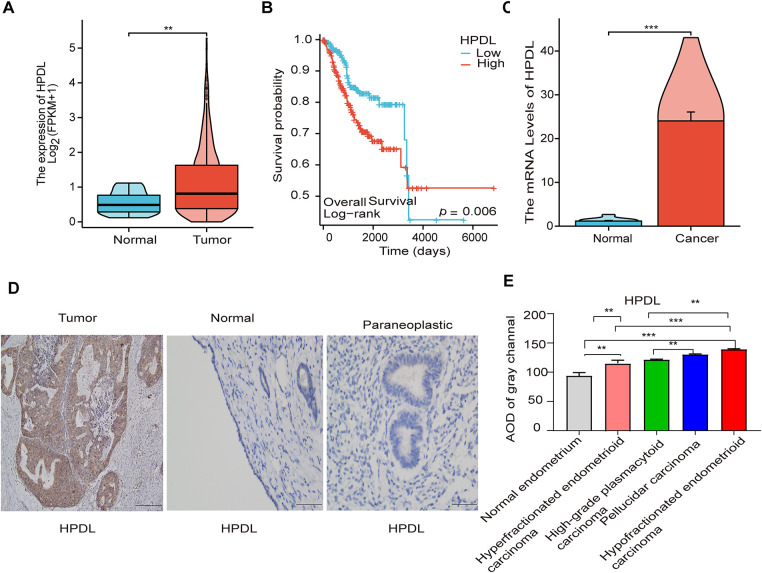
HPDL was significantly highly expressed in EC tissues. (**A**) Expression of HPDL in cancerous and normal tissues within the TCGA database; (**B**) Prognostic analysis of HPDL within the TCGA database. (**C**) Expression of HPDL mRNA in cancerous tissues and normal endometrium; (**D**) Expression of HPDL in EC tissues, normal endometrial tissues, and paracarcinomatous tissues. Scale bar is 100 μm; (**E**) Expression of HPDL in endometrial cancers of different pathologic types: Normal endometrium; Hyperfractionated endometrioid carcinoma; High-grade plasmacytoid carcinoma; Clear cell carcinoma; Pellucidar carcinoma; Hypofractionated endometrioid carcinoma. The scale bar represents 100 μm, and the black arrows in the figure indicate sites of positive expression of HPDL in the tissues, **signifying *p* < 0.01, and ***signifying *p* < 0.001

The analysis of the TCGA + GTEx database revealed that HPDL gene expression levels were significantly higher in endometrial carcinoma (EC) patient tissues compared to both normal and endometrial carcinoma-adjacent non-cancerous tissues (Fig. 2A). The HPDL gene correlates with poor prognosis in endometrial carcinoma (EC) patients (Fig. 2B). We subsequently validated HPDL expression in EC development. Compared to normal tissue and adjacent non-cancerous tissue, qPCR results confirmed significant overexpression of the HPDL gene in EC tissue (Fig. 2C). Immunohistochemistry revealed high HPDL expression in EC patient tissues (Fig. 2D). Furthermore, HPDL expression was higher in poorly differentiated endometrioid carcinomas than in well-differentiated endometrioid carcinomas. Both clear cell carcinomas and serous carcinomas exhibited significantly higher HPDL expression than normal endometrium and well-differentiated endometrioid carcinomas (Fig. 2E).

### 3.3 Knockdown of HPDL Reduces the Level of Histone Lactylation in EC Cells

**Figure 3:**
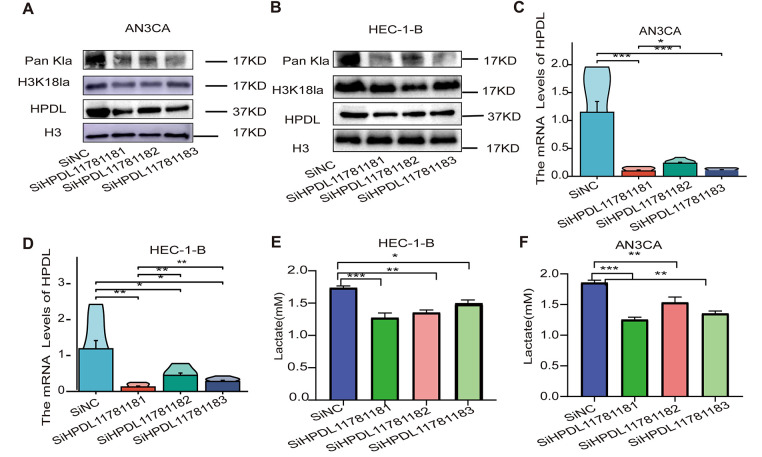

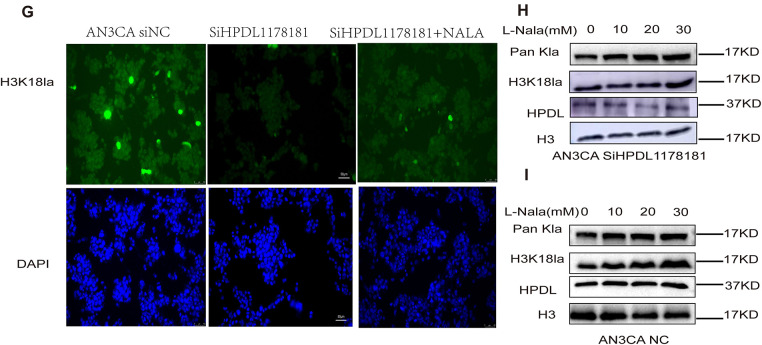
Knockdown of HPDL decreased the level of histone lactonization modification in EC cells. (**A**) Western blot (WB) analysis confirming the expression of histone lactate dehydrogenase (HLD) antibodies in AN3CA cells with HPDL knockdown; (**B**) WB analysis confirming the expression of HLD antibodies in HEC-1-B cells with HPDL knockdown; (**C**) Quantitative polymerase chain reaction (qPCR) analysis of HPDL expression following triple-sequence plasmid knockdown in AN3CA cells; (**D**) qPCR analysis of HPDL expression following triple-sequence plasmid knockdown in HEC-1-B cells; (**E**) Measurement of lactate content in AN3CA cells subsequent to HPDL knockdown; (**F**) Measurement of lactate content in HEC-1-B cells subsequent to HPDL knockdown. (**G**) Green fluorescence signifies HPDL protein expression, while blue fluorescence indicates immunofluorescence staining of cell nuclei; scale bar is 50 μm; (**H**) Expression levels of HPDL, Pan Kla, and H3K18la after the addition of varying concentrations of sodium lactate to AN3CA Si HPDL knockdown cell lines; scale bar represents 50 μm. Expression levels of HPDL, Pan Kla, and H3K18la in AN3CA Si HPDL knockdown cell lines with the addition of varying concentrations of sodium lactate; scale bar represents 50 μm; (**I**) Expression levels of HPDL, Pan Kla, and H3K18la after the addition of varying concentrations of sodium lactate to AN3CA normal cell lines. *indicates *p* < 0.05, **indicates *p* < 0.01, ***indicates *p* < 0.001

Western blot analysis confirmed that SiHPDL11781181 (We constructed three HPDL gene knockdown plasmids, designated SiHPDL11781181, SiHPDL11781182, and SiHPDL11781183, to determine which sequence yields the most effective knockdown) resulted in the lowest HPDL protein expression level after transient transfection in AN3CA and HEC-1-B cells, and SiHPDL11781181 also exhibited the highest knockdown efficiency (Fig. 3A,B). Thus, we selected SiHPDL11781181 for lentiviral packaging to establish EC cell lines with stable knockdown. Furthermore, qPCR revealed that HPDL mRNA expression was lowest in the SiHPDL groups of AN3CA and HEC-1-B cells (Fig. 3C,D), and we observed a significant reduction in the lactate content in AN3CA and HEC-1-B cell lines with transient HPDL knockdown (Fig. 3E,F). Western blot analysis of the Pan Kla and H3K18la levels in HPDL-knockdown AN3CA and HEC-1-B cells indicated that the levels of Pan Kla and H3K18la were significantly reduced by HPDL knockdown (Fig. 3A,B). suggesting that knockdown of HPDL significantly inhibited histone lactylation. After L-NaLa treatment of normal and HPDL knockdown AN3CA cell lines, the expression of H3K18la was detected by immunofluorescence staining, with green fluorescence indicating H3K18la and blue fluorescence indicating the nuclear marker DAPI. The level of H3K18la was significantly reduced in the HPDL knockdown group compared with the NC group, and the level of H3K18la was significantly elevated after the addition of 30 mmol/L lactate (Fig. 3G). Different concentrations (0, 10, 20, and 30 mmol/L) of sodium lactate were added to NC and SiHPDL11781181 AN3CA cells, and measurement of the Pan Kla and H3K18la levels in the cells in both groups confirmed that the histone lactylation level in endometrial carcinoma cells was elevated, but there was no significant increase in HPDL expression (Fig. 3H,I). The above results indicated that sodium lactate supplementation could significantly increase the histone lactylation level and that the lactate-induced increase in the histone lactylation level did not affect the expression of HPDL.

### 3.4 The Histone Lactylation Level Is Elevated in EC Cells after the Overexpression of HPDL

**Figure 4:**
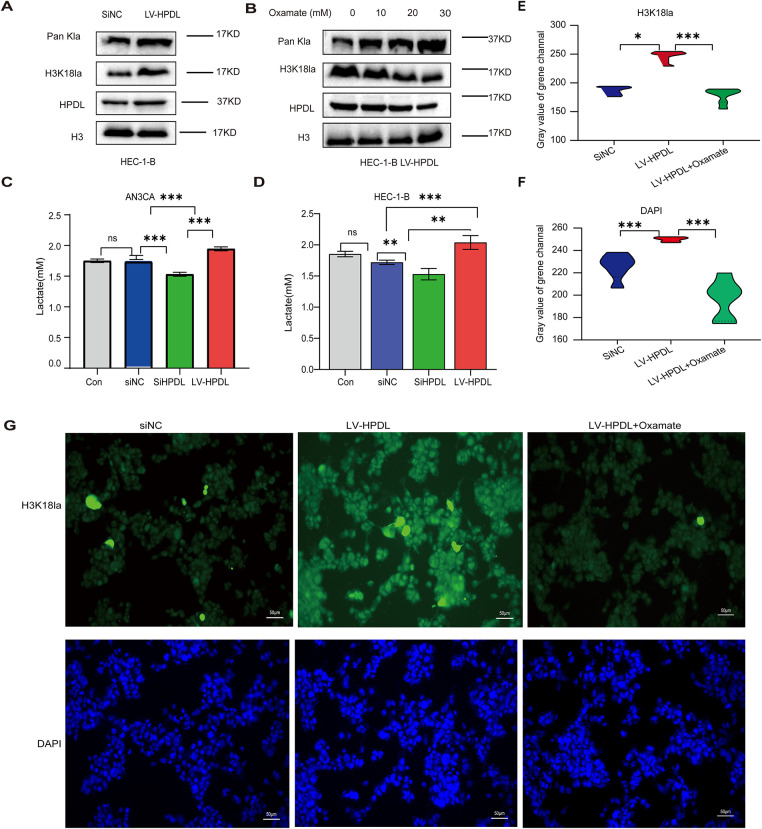
The level of histone lactylation was elevated in EC cells overexpressing HPDL (**A**) Expression levels of HPDL, Pan Kla, and H3K18la following the overexpression of HPDL in the HEC-1-B cell line; (**B**) Expression levels of HPDL, Pan Kla, and H3K18la after treating the HEC-1-B cell LV-HPDL with varying concentrations of sodium oxalate; (**C**) Overexpression and knockdown strains of AN3CA; (**D**) Lactate levels in HEC-1-B overexpression and knockdown strains; (**E**) Immunofluorescence of AN3CA cells for quantifying H3K18la immunofluorescence across different groups; (**F**) Immunofluorescence of AN3CA cells for quantifying DAPI immunofluorescence across different groups; (**G**) Immunofluorescence of AN3CA cells for quantifying the expression of H3K18la and DAPI, scale bar is 50 μm; *indicating *p* < 0.05, **indicating *p* < 0.01, ***indicating *p* < 0.001, and ns indicating no statistical significance

We transfected AN3CA and HEC-1-B cells with the lentiviral overexpression vector LV-HPDL (28880-1) to construct a cell line with stable HPDL overexpression. Following puromycin screening, once the cells had stabilized, Western Blot (WB) analysis revealed a significant increase in HPDL expression in the HPDL-overexpressing HEC-1-B cell line, as well as a notable increase in the signal of the anti-Pan Kla antibody in the HPDL-overexpressing group. The signal of the anti-H3K18la antibody was also elevated (Fig. 4A). The histone lactylation inhibitor sodium oxamate was added to HPDL-overexpressing HEC-1-B cells at concentrations of 0, 10, 20, and 30 mmol. WB analysis showed that the signals of the anti-Pan Kla and anti-H3K18la antibodies were significantly reduced in these cells, although HPDL expression remained unchanged (Fig. 4B). Lactate content assays indicated that lactate levels were significantly higher in HPDL-overexpressing cells but markedly lower in HPDL-knockdown cells (Fig. 4C,D). Immunofluorescence analysis of AN3CA cells demonstrated that H3K18la and Pan Kla levels were elevated in the HPDL overexpression group, and the addition of oxamate to the HPDL-overexpressing cells significantly decreased the H3K18la and DAPI signals (Fig. 4E–G). This confirmed that oxamate could inhibit histone lactylation in endometrial carcinoma cells and that the addition of oxamate did not inhibit HPDL expression after histone lactylation was suppressed.

### 3.5 HPDL Promotes the Proliferation of EC Cells

**Figure 5:**
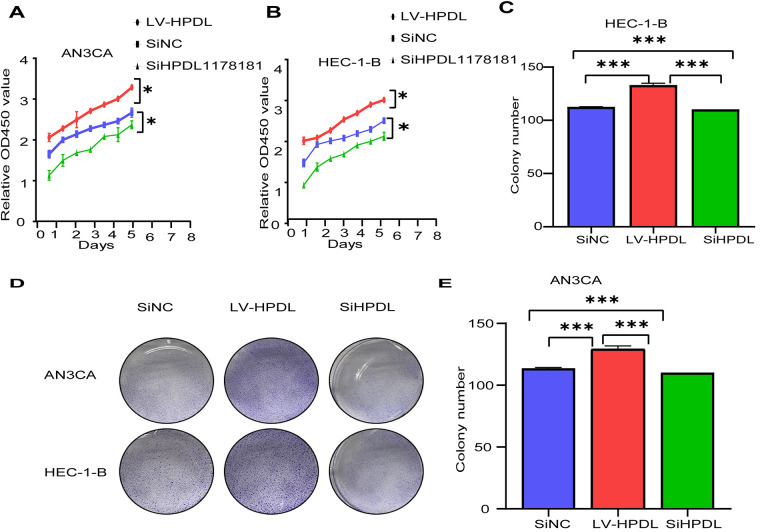

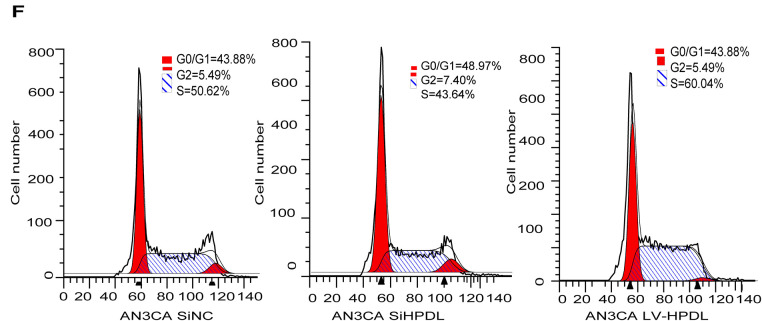
The expression of HPDL promotes the proliferation of EC cells and affects the cell cycle. (**A**) CCK-8 profiles in AN3CA cells; (**B**) CCK-8 profiles in HEC-1-B cells; (**C**) Quantitative results of plate cloning assay of HEC-1-B cells; (**D**) Plate cloning assay of AN3CA and HEC-1-B cells; (**E**) Quantification results of plate cloning of AN3CA cells; (**F**) AN3CA cell cycle assay, *indicating *p* < 0.05 and ***indicating *p* < 0.001

To verify the effect of HPDL expression on cell proliferation, we laid 96-well plates in the SiNC, SiHPDL, LVHPDL groups of AN3CA and HEC-1-B cells, with 5 wells in each group, and detected the absorbance of CCK-8 at different times to plot the curves as shown in the graphs (Fig. 5A,B), which showed that the cell proliferation rate of the LVHPDL group was significantly increased compared to that of the SiNC group. The cell proliferation of SiHPDL group was significantly decreased. The SiNC, SiHPDL, and LVHPDL groups of AN3CA and HEC-1-B were laid in 6-well plates, and plate cloning experiments were performed, and the number of cell clones was detected after 2 weeks of incubation, and the results showed that the number of cell clones of the LVHPDL group > SiNC group > SiHPDL group, and the difference was statistically significant (Fig. 5C–E), which showed that the cell proliferation ability of cells in the HPDL overexpression group was enhanced, and the cell proliferation ability of cells in the knockdown group was decreased, and the expression of HPDL could promote the proliferation of endometrial cancer cells. The effect of HPDL expression on the cell cycle was detected by flow cytometry, and the results showed that the proportion of S phase, which reflects the proliferation ability of cells, increased in the LVHPDL group, and the proportion of G0/G1 decreased; whereas, the proportion of S phase decreased and the proportion of G0/G1 increased after the knockdown of HPDL (Fig. 5F), suggesting that the expression of HPDL may regulate the cycle.

### 3.6 HPDL Promotes the Migration and Invasion of EC Cells

**Figure 6:**
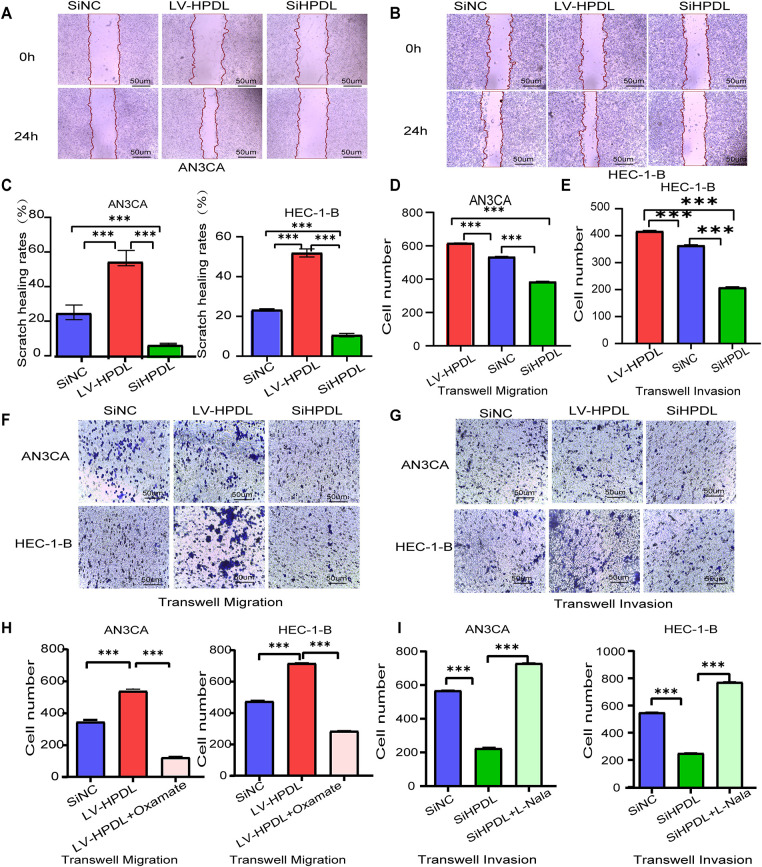
HPDL promotes the migration and invasion of EC cells. (**A**) Cell scratch assay of the normal, overexpression and knockdown groups of the HPDL gene in AN3CA cells, scale bar is 50 μm; (**B**) Cell scratch assay of the normal, overexpression and knockdown groups of the HPDL gene in HEC-1-B cells; (**C**) AN3CA and HEC-1-B cells with the normal, overexpression and knockdown groups of the HPDL gene in the quantitative histograms of cell scratch assay cells; (**D**) quantitative histograms of Transwell migration assay of AN3CA cells; (**E**) quantitative histograms of Transwell migration assay of HEC-1-B cells; (**F**) Transwell migration assay of AN3CA and HEC-1-B cells, scale bar is 50 μm; (**G**) AN3CA and HEC-1-B cells Transwell invasion assay; (**H**) AN3CA and HEC-1-B cell HPDL overexpression Transwell invasion assay; (**I**) Transwell invasion assay with HPDL knockdown in AN3CA and HEC-1-B cells, ***indicating *p* < 0.001

For further elucidation of the effect of HPDL on the biological phenotype of endometrial cancer (EC) cells, this study examined the migratory ability of AN3CA and HEC-1-B cells after HPDL overexpression and knockdown by scratch assay. The results showed that the scratch healing rate was significantly higher in the LV-HPDL group compared with the SiNC group, while it was significantly lower in the SiHPDL group (Fig. 6A–C). Transwell migration assay showed that the number of membrane-penetrating cells was significantly higher in the LV-HPDL group compared with the SiNC group in both AN3CA and HEC-1-B cells, while it was significantly lower in the SiHPDL group (Fig. 6D,F), confirming that HPDL significantly enhanced the migration ability of EC cells. Transwell invasion assay showed that the number of membrane-penetrating cells was significantly increased in the LV-HPDL group compared to the SiNC group, whereas it was significantly decreased in the SiHPDL group (Fig. 6E,G), confirming that HPDL promotes the invasive ability of EC cells.

We added the histone lactylation inhibitor sodium oxalate to the HPDL overexpression group and observed the change in cell invasion in the HPDL overexpression group after histone lactylation inhibition. 6-well plate Transwell invasion experiments were performed by establishing the SiNC group, LV-HPDL group and LV-HPDL+oxalate group of AN3CA and HEC-1-B cells, and found that the LV-HPDL group had a significant increase in the number of membrane-penetrating cells compared with the SiNC group, while the LV-HPDL+oxalic acid group had a significant decrease compared with the LV-HPDL group (Fig. 6H). The results confirmed that histone lactylation modification inhibited cell migration and invasion induced by HPDL overexpression, suggesting that HPDL overexpression may promote migration and invasion of EC cells by regulating histone lactylation.

We added the histone lactylation enhancer sodium lactate to the HPDL knockdown group to observe the alteration of cell invasion in the HPDL overexpression group after histone lactylation inhibition. 6-well plate Transwell invasion experiments were performed by establishing the SiNC group, the SiHPDL group and the SiHPDL+sodium lactate group of AN3CA and HEC-1-B cells, and the results showed that the number of membrane-penetrating cells in the SiHPDL group was significantly fewer than the SiNC group, whereas the SiHPDL+sodium lactate group had significantly more than the SiHPDL group (Fig. 6I). These confirmed that promotion of histone lactylation reversed the decrease in cell migration and invasion ability caused by HPDL knockdown, further suggesting that HPDL knockdown may inhibit the migration and invasion of EC cells by regulating histone lactylation.

### 3.7 HPDL Promotes Histone Lactylation in EC Cells by Upregulating LDHA/LDHB Expression

We downloaded genes that were positively correlated with HPDL in EC from the TCGA database. A total of 544 genes had correlation coefficients greater than 0.3, and 466 of these genes were found to be differentially expressed in EC through expression analysis. The differentially expressed genes underwent GO/KEGG enrichment analyses, which indicated that these genes were primarily enriched in fatty acid metabolism, mitochondrial metabolism, carbon metabolism, and central carbon metabolism in cancer (Table 1).

**Table 1:** GO/KEGG analysis of HPDL-related genes

Ontology	Description	Gene ratio	*p*-value
BP	DNA-template DNA replication	29/421	1.96e−18
BP	Nuclear chromosome segregation	36/421	4.57e−17
BP	Chromosome segregation	39/421	1.07e−16
BP	DNA replication	35/421	1.42e−16
CC	Chromosomal region	41/432	1.03e−17
CC	Spindle	36/432	1.05e−12
CC	Chromosome, centromeric region	26/432	6.93e−12
CC	Condensed chromosome	24/432	2.58e−09
MF	Catalytic activity, acting on DNA	30/432	9.17e−15
MF	Single-stranded DNA helicase activity	11/432	1.1e−12
MF	ATP-dependent activity, acting on DNA	17/432	2.62e−10
MF	DNA helicase activity	14/432	1.08e−09
MF	Helicase activity	19/432	4.23e−09
KEGG	DNA replication	14/204	5.55e−14
KEGG	Cell cycle	20/204	2.96e−11
KEGG	Base excision repair	9/204	7.3e−08
KEGG	Ribosome biogenesis in eukaryotes	13/204	2.87e−06
KEGG	Central carbon metabolism in cancer	6/204	1.6e−05

Note: HPDL, 4-hydroxyphenylpyruvate dioxygenase-like; GO, Gene Ontology; KEGG, Kyoto Encyclopedia of Genes and Genomes; BP, Biological Process; CC, Cellular Component; MF, Molecular Function.

**Figure 7:**
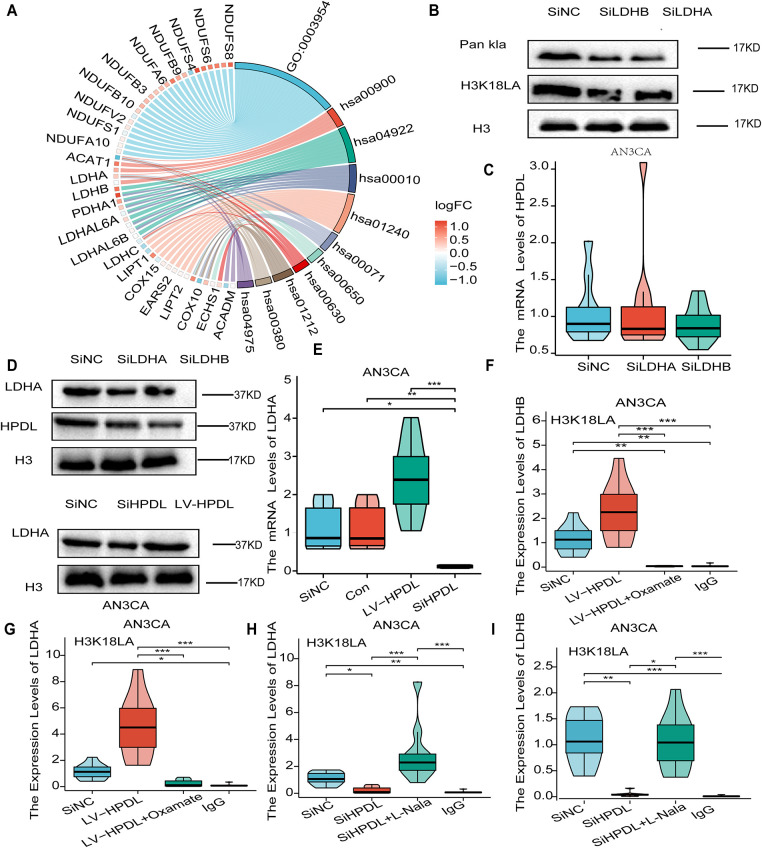
HPDL promotes histone lactylation levels in EC cells by upregulating LDHA/LDHB expression. (**A**) Chordal graph of GO/KEGG analysis; (**B**) Western blot (WB) demonstrates detection of Pan Kla, H3K18la antibody expression after LDHA and LDHB inhibition; (**C**) Reverse transcription-quantitative polymerase chain reaction (qPCR) showing the expression of HPDL in AN3CA cells with SiNC, SiLDHA, and SiLDHB; (**D**) WB demonstration of fluorescence transfection efficiency and LDHA/LDHB and HPDL expression in AN3CA cells with SiNC, SiLDHA, and SiLDHB; (**E**) qPCR of AN3CA cells with decreased LDHA/LDHB expression in the SiHPDL group, and decreased LDHA/LDHB expression in the LVHPDL group, where LDHA/LDHB expression increased; (**F)** H3K18la expression in the LDHA promoter region in AN3CA cells with SiNC, LV-HPDL, and LV-HPDL+Oxmate; (**G**) H3K18la expression in the LDHB promoter region in AN3CA cells with SiNC, LV-HPDL, and LV-HPDL+Oxmate; (**H**) H3K18la expression in the LDHA promoter region in AN3CA cells with SiNC, SiHPDL, and SiHPDL+L-NaLa; (**I**) H3K18la expression in the LDHB promoter region in AN3CA cells with SiNC, SiHPDL, and SiHPDL+L-NaLa; *indicates *p* < 0.05, **indicates *p* < 0.01, ***indicates *p* < 0.001

Within these processes, the downstream genes of HPDL in the carbon metabolism pathway of cancer cells are LDHA and LDHB. LDHA and LDHB participate in glycolytic metabolism, and their metabolite is lactate; therefore, since lactate serves as a substrate for histone lactylation, LDHA and LDHB may play a crucial role in histone lactylation. Consequently, we concentrated on this pathway and investigated the relationship between HPDL and its associated genes, LDHA/LDHB, as well as their regulatory roles in histone lactylation within endometrial cancer. The pathway map associated with the GO/KEGG analyses is depicted in (Fig. 7A,B. Western blot (WB) detection revealed that after knocking down LDHA and LDHB, quantitative PCR results showed no significant changes in HPDL mRNA expression levels (Fig. 7C), and HPDL protein expression did not exhibit significant differences in *Parazacco spilurus* subsp. spilurus (Fig. 7D), suggesting that there was no significant alteration in HPDL expression subsequent to substantial inhibition of LDHA/LDHB expression. The mRNA and protein levels of LDHA and LDHB in AN3CA cells were examined across three groups: the HPDL overexpression group, the HPDL knockdown group, and the SiNC group. Western blot (WB) analysis revealed that the expression of LDHA/LDHB was significantly downregulated in the SiHPDL group and significantly upregulated in the LV-HPDL group (Fig. 7D). Quantitative polymerase chain reaction (qPCR) analysis indicated that LDHA/LDHB mRNA expression was decreased in the SiHPDL group and significantly increased in the LV-HPDL group (Fig. 7E). These results suggest that knockdown of HPDL significantly downregulates LDHA/LDHB expression, while overexpression of HPDL significantly upregulates it.

Following the knockdown of LDHA and LDHB, Western blot analysis revealed that the signals from the anti-Pan Kla and anti-H3K18la antibodies were significantly suppressed. This indicates that the knockdown of LDHA/LDHB markedly inhibited histone lactylation in EC cells (Fig. 7B). The levels of H3K18la in the promoter regions of LDHA and LDHB were elevated in the HPDL-overexpressing AN3CA cell line and subsequently decreased upon the addition of oxalate (Fig. 7F,G). Supplementation with sodium oxamate led to a reduction in the H3K18la level in the LDHA promoter region (Fig. 7F,G). The levels of H3K18la in the LDHA and LDHB promoter regions were diminished in the AN3CA HPDL-knockdown cell line, and the introduction of sodium lactate increased the levels of H3K18la in the promoter regions of both LDHA and LDHB (Fig. 7H,I). These results confirm that HPDL regulates the expression of LDHA and LDHB through histone lactylation at H3K18. In summary, these findings indicated that HPDL regulated the expression of LDHA/LDHB; however, knockdown of LDHA/LDHB had no significant effect on the expression of HPDL. Additionally, knockdown of LDHA/LDHB significantly inhibited histone lactylation in AN3CA cells.

### 3.8 In Vivo Experiments Confirmed That HPDL Promotes Endometrial Cell Proliferation and Growth

**Figure 8:**
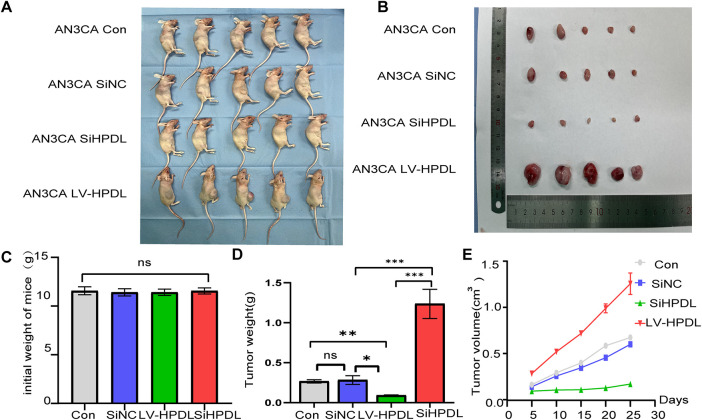
The expression of HPDL promotes tumor growth. (**A**,**B**) Mouse macroscopic and tumor phenotypes post-euthanasia; (**C**) Initial body weight of mice; (**D**) Tumor body weight; (**E**) Tumor volume growth curves. ns denotes *p* > 0.05, *denotes *p* < 0.05, **denotes *p* < 0.01, and ***denotes *p* < 0.001

We randomly divided 4-week-old BALB/c nude mice into four groups of five mice each. Fig. 8A,B demonstrated the macroscopic morphology of mice and tumors after euthanasia, and there was no significant difference in the initial body weights of the mice (Fig. 8C). After the isolation and quarantine period, we established SiNC group, AN3CA control group, HPDL knockdown group, and HPDL overexpression group. After culturing the cells by passaging to an optimal growth state, the mice were inoculated at a density of 5 × 10^6^ cells per mouse. The tumor volume of the LV-HPDL overexpression group was significantly larger than that of the SiNC group and AN3CA control group, whereas the tumor volume of the HPDL knockdown (SiHPDL) group was significantly smaller than that of the SiNC group, and the difference in tumor weight was statistically significant (Fig. 8D). The tumor growth rates and tumor sizes in the HPDL overexpression group were significantly higher than those of the SiNC group (Fig. 8D). The tumor growth rates and tumor sizes in the HPDL overexpression group were statistically significant. It had a significantly higher tumor growth rate and tumor size than the SiNC group and AN3CA control group, while the HPDL knockdown (SiHPDL) group was significantly lower than these two control groups (Fig. 8E). These findings indicated that HPDL overexpression significantly promoted, whereas HPDL knockdown significantly inhibited, tumor growth in nude mice.

## 4 Discussion

### 4.1 HPDL Has the Potential to Predict the Prognosis of EC Patients

Endometrial carcinomas (ECs) represent a group of malignant epithelial tumors arising from the endometrium and rank among the most prevalent gynecological malignancies [11]. While early-stage EC patients generally exhibit favorable prognoses, those with advanced, recurrent, or metastatic disease face significantly poorer outcomes. Given the limited efficacy of radical surgery in such cases [12], there is an urgent clinical need to identify novel molecular biomarkers for diagnosis, prognostication, and targeted therapy.

The HPDL gene, a recently characterized single-exon gene encoding a mitochondrial-localized protein [13], has emerged as a candidate of interest. Although initially implicated in tyrosine metabolism, metabolic profiling of HPDL-mutant cells and murine models revealed no tyrosine pathway alterations but significant dysregulation in other metabolic compartments. This phenotype aligns with HPDL loss-of-function causing a distinct pediatric neurometabolic mitochondrial disorder [14]. Critically, oncogenic roles for HPDL have been reported: Ye et al. demonstrated that HPDL overexpression promotes pancreatic ductal adenocarcinoma (PDAC) tumorigenesis *in vitro*, whereas its suppression inhibits proliferation and clonogenicity [10].

Extending these findings to EC: Through constructing a lactate-related prognostic model for endometrial cancer, our prior work identified HPDL as a high-risk gene. Subsequent validation via Western blot and quantitative PCR analyses of clinical specimens confirmed significantly elevated HPDL expression in EC tissues compared to normal endometrium. Critically, this overexpression correlated with adverse clinical outcomes, underscoring HPDL’s potential as both a prognostic biomarker and therapeutic target in EC.

### 4.2 Elevated Levels of Histone Lactylation in Endometrial Cancer

Histones are proteins that consist of a nucleosome core wrapped in DNA and contain numerous posttranslational modifications in the N-terminal tail. These modifications can regulate physiological processes closely related to gene expression, such as RNA transcription, DNA replication, and DNA damage repair, either by altering the structure of the nucleosome or by recruiting nonhistone proteins [15]. Many of the canonical types of histone posttranslational modifications, such as methylation and acetylation, have long been recognized as important in the pathogenesis of a range of other diseases, including cancer [16]. Researchers have identified lactylation as a novel epigenetic modification of histone lysine residues, and detected 28 lactylation sites in core histones across human and mouse cells [17]. Hypoxia and bacterial stress induce lactate production via glycolysis, and lactate in turn functions as a precursor to stimulate histone lactylation [18]. Subsequently, an increasing number of studies have shown that tumours, inflammation, metabolism, and hypoxia are associated with the regulation of histone lactylation, and Feng et al. [19] reported that lactate induced histone lactylation at the promoters of profibrotic genes in macrophages, consistent with the increase in this epigenetic modification in fibrotic lungs. This lactate-induced histone lactylation and fibrotic gene expression were mediated by p300, as evidenced by their reductions in p300-deactivated macrophages [20]. Yang et al. [21] demonstrated that increased glycolytic activity could induce endometrial histone lactylation, a newly identified histone modification, remodelling a novel function in uterine tolerance. The results from *in vitro* and *in vivo* models support an important role for lactate in inducing endometrial H3K18la and regulating redox homeostasis and the apoptotic balance to ensure successful embryo implantation. Our group previously confirmed that the level of histone lactylation is elevated in EC tissues.

### 4.3 HPDL Expression Drives Proliferation and Cell Cycle Progression in EC

Dysregulation of cell cycle machinery is a hallmark of tumorigenesis and represents a rational therapeutic target [22]. Notably, cell cycle pathways intersect with cancer metabolism and immune evasion mechanisms [23], suggesting that targeting these pathways may simultaneously inhibit proliferation, reverse metabolic reprogramming, and restore immune surveillance. While small-molecule inhibitors (SMIs) and proteolysis-targeting chimeras (PROTACs) have shown promise in modulating cell cycle proteins [24], recent work also implicates cytoplasmic DNA sensors (e.g., cGAS-STING) in cell cycle control and tumor suppression [25].

In this study, we examined the migration ability of AN3CA and HEC-1-B cells after HPDL overexpression and knockdown by scratch assay, and confirmed that HPDL could promote the invasion ability of EC cells. The histone lactylation inhibitor sodium oxalate was added to the HPDL overexpression group to observe the alteration of cell invasion in the HPDL overexpression group after the inhibition of histone lactylation. The results confirmed that histone lactylation modification could inhibit HPDL overexpression-induced cell migration and invasion, suggesting that HPDL overexpression might promote the migration and invasion of EC cells through the regulation of histone lactylation. The histone lactylation enhancer sodium lactate was added to the HPDL knockdown group to observe the alteration of cell invasion in the HPDL knockdown group after the inhibition of histone lactylation. These confirmed that the promotion of histone lactylation reversed the decrease of cell migration and invasion caused by HPDL knockdown, which further suggests that HPDL knockdown may inhibit the migration and invasion of EC cells through the modulation of histone lactylation.

HPDL drives EC progression by accelerating cell cycle transition and promoting metastasis via histone lactylation. Targeted inhibition of HPDL represents a promising therapeutic strategy for endometrial cancer.

### 4.4 HPDL Upregulates LDHA/LDHB Expression and Promotes Histone Lactylation in EC Cells

Tumor cells exhibit high metabolic plasticity and can engage in anaerobic glycolysis and lactate production under hypoxic conditions [26]. The enzymes responsible for the reversible conversion of pyruvate to lactate include LDHA and LDHB [27]. LDHA has a high affinity for pyruvate and preferentially converts it to lactate and NADH to NAD+ under anaerobic conditions, while LDHB has a high affinity for lactate and preferentially converts it back to pyruvate in the presence of sufficient oxygen, also converting NAD+ to NADH. In addition to their undisputed roles in tumor cell metabolism and adaptation to adverse environmental or cellular conditions, LDHA and LDHB are also involved in histone lactylation [28]. Nian et al. [29] reported that LDHA expression is upregulated in human non-small cell lung cancer (NSCLC) tissues and correlates with poor disease prognosis. LDHA further increases histone lactylation and activates the expression of HK-1 and IDH3G, leading to metabolic disorders and tumorigenesis in lung cancer cells. Tang et al. [30] reported that the levels of LDHA, intracellular lactate, and histone lactylation progressively increase during osteogenic differentiation. Downregulation of LDHA affects the formation of mineralized nodules and ALP activity; knockdown of LDHA decreases the enrichment of histone lactylation at the JunB promoter, and exogenous lactate treatment reverses this effect [31].

### 4.5 HPDL Expression Regulates Histone Lactylation in Endometrial Cancer

In this study, we revealed for the first time the critical regulatory role of hydroxy acid dehydrogenase (HPDL) on histone lactate modification in endometrial cancer (EC) by functional experiments. Our results show that transient knockdown of HPDL expression in two endometrial cancer cell lines, AN3CA and HEC-1-B, resulted in a significant decrease in intracellular lactate content, overall protein lactate level (Pan Kla), and specific histone H3K18 lactate (H3K18la) modification. This finding directly links HPDL, a metabolic enzyme, to the emerging epigenetic modification of histone lactylation, providing a novel “metabolic-epigenetic” perspective for understanding the mechanisms of endometrial cancer progression.

At the core of our findings is the demonstration that HPDL is a key upstream factor in maintaining high lactate levels in cancer cells, and is thought to catalyze the oxidative decarboxylation of 2-hydroxy acids (e.g., 2-hydroxyglutaric acid) in the mitochondria, a process that is potentially linked to energy metabolism and lactate metabolism networks [32]. Although its exact function is still being explored, our data clearly show that knockdown of HPDL directly contributes to the depletion of the intracellular lactate pool. This strongly suggests that in EC cells, HPDL may act as a “gatekeeper” of intracellular lactate levels through its metabolic function by influencing a key aspect of the glycolytic process: lactate production or lactate conversion. This finding provides a new example of how tumor cells finely regulate their metabolite concentrations to drive downstream signaling pathways.

The decrease in intracellular lactate levels directly led to a widespread inhibition of histone lactate modification. This causal relationship perfectly corroborates the groundbreaking study by Prof. Yi Zhang’s team in 2019, which for the first time suggested that lactate could serve as a precursor substrate for histone lactonization, thereby directly coupling cellular metabolism to epigenetic regulation [17]. As a novel epigenetic modification, histone lactylation has been shown to activate the expression of genes involved in tumorigenesis, Warburg effect, and immune regulation, among other related genes, by altering chromatin structure and recruiting transcriptional regulatory complexes. Thus, the down-regulation of Pan Kla and H3K18la that we observed is not an isolated biochemical phenomenon, but rather implies that HPDL knockdown may have triggered the reprogramming of a series of pro-oncogenic transcriptional programs by decreasing the level of lactonization.

Of particular interest is the downregulation of H3K18la. It has been shown that H3K18la is an active transcriptional activation marker on gene promoters. In macrophages, it was shown to be involved in the activation of M2-type polarization-related genes. In the context of cancer, high levels of H3K18la are strongly associated with malignant progression of several tumors [33]. Our results suggest that HPDL-driven lactate production is required to maintain high levels of H3K18la in endometrial cancer. This implies that HPDL may specifically activate a critical set of genes that drive EC cell proliferation, migration, or stemness maintenance through upregulation of H3K18la, thereby exerting its oncogenic function. Future combined ChIP-seq (chromatin immunoprecipitation sequencing) and RNA-seq (transcriptome sequencing) analyses will hopefully reveal precisely which oncogenes are dependent on the HPDL-H3K18la axis for their activation.

From a clinical translational perspective, our study has dual implications. First, HPDL and its regulated histone lactylation levels might serve as novel prognostic biomarkers for EC patients. High levels of HPDL/H3K18la may portend greater metabolic activity and poorer clinical prognosis. Second, and more importantly, HPDL shows great promise as a potential therapeutic target. Compared with the traditional strategy of targeting key enzymes of glycolysis (e.g., LDHA), targeting upstream HPDL may be more specific and effective in cutting off the source of lactate, thus not only inhibiting the energy supply of tumor cells, but also reversing their pro-cancer epigenetic status, thus achieving the therapeutic effect of “killing two birds with one stone”. In addition, in view of the inhibitory effect of lactate on immune cells in the tumor microenvironment [34], targeting HPDL may also enhance the efficacy of existing immunotherapies by decreasing lactate levels and remodeling the immune microenvironment.

Of course, this study still has some limitations. The current data were mainly derived from *in vitro* cell line models, and further validation in *in vivo* animal models (e.g., transplantation tumor models) and a wider range of EC clinical samples is needed. In addition, the specific molecular mechanisms by which HPDL regulates lactate levels (e.g., what are its most critical substrates and how does it cross-talk with the glycolytic pathway) remain to be elucidated in depth.

## 5 Limitations

The limitation of this study is that our team focused on an *in vitro* EC cell model (AN3CA/HEC-1-B) and *in vitro* cellular experiments, as well as a clinical case study, which would have been better with more cases. Limitations of the evidence for the mechanism of the direct interaction of HPDL-LDHA/LDHB. “Although our *in vivo* data establish HPDL as a viable therapeutic target, they do not resolve whether the mechanisms identified *in vitro* (e.g., mechanism) underpin these phenotypes. Future studies using [specific tools] will bridge this gap”.

## 6 Conclusion

This study found that inhibiting the expression of LDHA or LDHB significantly suppressed histone lactylation in endometrial cancer. However, such inhibition did not notably affect HPDL expression. Conversely, suppressing HPDL expression led to a significant decrease in LDHA/LDHB expression, and overexpression of HPDL markedly downregulated LDHA/LDHB expression. Consequently, we concluded that HPDL can facilitate histone lactylation in EC by upregulating LDHA/LDHB.

## Data Availability

All data generated or analyzed during this study are included in this published article.

## Appendix A

**Table A1:** Antibodies & detection reagents

Reagent name (English)	Company	Cat. No.
Prestained protein marker (Dual Color)	Yeasen	WJ102
HRP-conjugated goat anti-rabbit IgG	Yeasen	LF102
HRP-conjugated goat anti-mouse IgG	PTMBIO	1401
Pan anti-lactyllysine antibody	Yeasen	LF101
H3K18la antibody	PTMBIO	1406
Histone H3 antibody	Abbkine	A01070
Goat serum	Abbkine	BMS005
MEM with NEAA	Procell	PM150410
Reagent name (English)	Company	Cat.No.
Fetal bovine serum (South America)	ExCell Bio	FSP500
Trypsin-EDTA solution	Biosharp	BL501A
PBS buffer	Biosharp	BL302A
Penicillin-streptomycin solution	Biosharp	BL505A
Cell freezing medium	New Cell Media	C40100
HEC-1-B cell line	Procell	CL-0100
AN3CA cell line	Procell	CL-0530
L-sodium lactate	Sigma	71718
Sodium oxamate	Absin	abs811929
Reverse transcription kit	Novoprotein	E047
qPCR Kit	Novoprotein	E096
TRIzol reagent	Invitrogen	1559602
Lactate assay kit	Biovision	K627
Skim milk blocking powder	Beyotime	P0216
4% Paraformaldehyde	Lanjieke	BL539A
Triton X-100	Solarbio	T8200
0.5M tris buffer (pH 6.8)	Sbjbio	BL-S012
Glycerol	Solarbio	G8190
10% SDS solution	Solarbio	S1010
β-Mercaptoethanol	Solarbio	M8210
Bromophenol blue	Solarbio	B8120
PAGE gel kit (12.5%)	Yeasen	PG114
Tris/Glycine/SDS running buffer (10X)	Yeasen	PS105S
Transfer buffer (10X)	Yeasen	PG109S
Methanol	Macklin	M813895
T25 cell culture flask	Greiner	690160
100mm culture dish	Greiner	664160
Cryovials (2 mL)	Greiner	123278
6-well plate	Kirgen	KG10006
0.2 mL PCR tubes	Kirgen	KG2331
50 mL centrifuge tubes	Kirgen	KG2811
15 mL centrifuge tubes	Kirgen	KG2611

**Table A2:** PCR primer sequence

Primer name	Sequence (5^′^ to 3^′^)		Number of bases
LDHA promoter region	Forward primer	TTGACCTACGTGGCTTGGAAG	21
LDHA promoter region	Forward primer	TTGACCTACGTGGCTTGGAAG	21
LDHA promoter region	Reverse primer	GGTAACGGAATCGGGCTGAAT	21
LDHB promoter region	Forward primer	TCTGTGACCGCCAATTCTAAGA	22
LDHB promoter region	Reverse primer	GCACCAGATTGAGCCGACTC	20
HPDL	Forward primer	ACGCGGTCTTTTTGGTGAAC	20
HPDL	Reverse primer	CCACGTCGAAGCACAGGTTT	20
LDHA	Forward primer	ATGGCAACTCTAAAGGATCAGC	22
LDHA	Reverse primer	CCAACCCCAACAACTGTAATCT	22
GAPDH	Forward primer	CTGGGCTACACTGAGCACC	19

**Table A3:** Statistical table of patients’ clinical data

Serial number	Pathology number	Hospitalization number	Age	Date of surgery	Vascular invasion	Lymph node	Grade	Pathological type	State
1	202157884	6719483001	68	2021.11.29	None	Negative	G1	Endometrioid carcinoma	Survival
2	202155551	6712864002	59	2021.11.16	None	Negative	G2	Endometrioid carcinoma	Survival
3	202155554	6712833001	79	2021.11.16	None	Negative	G3	Poorly differentiated endometrial carcinoma, suggestive of high-grade serous carcinoma	Death
4	202154127	6708581001	46	2021.11.08	None	Negative	G2	Endometrioid carcinoma	Survival
5	202149437	6697214001	56	2021.10.12	None	Negative	G3	Endometrioid carcinoma	Survival
6	202144507	6685737001	58	2021.09.08	None	Negative	G2	Endometrioid carcinoma	Survival
7	202142823	6678397001	66	2021.08.30	None	Negative	G2	Metastasis of Pellucidar carcinoma to the ovary	Survival
8	202141554	6675964001	56	2021.08.23	None	Negative	G2	Endometrioid carcinoma	Survival
9	202141574	6678227001	55	2021.08.21	None	Negative	G3	Endometrioid carcinoma	Survival
10	202141234	6676420001	31	2021.08.19	None	Negative	G2	Endometrioid carcinoma	Survival
11	202139437	6672385	59	2021.08.15	None	Negative	G3	Hypofractionated endometrioid carcinoma	Survival
12	202139191	6672056001	56	2021.08.10	None	Negative	G2	Endometrioid carcinoma	Survival
13	202133818	6659514	47	2021.07.14	None	Negative	G2	Endometrioid carcinoma	Survival
14	202133823	6659321001	53	2021.7.14	None	Negative	Unreported	Endometrioid carcinoma	Survival
15	202119873	6624550001	49	2021.04.27	None	Negative	G3	Hypofractionated endometrioid carcinoma	Survival
16	202119202	6622279001	59	2021.04.23	None	Negative	G1	Endometrioid carcinoma	Survival
17	202116750	6616935001	56	2021.04.12	None	Negative	G2	Endometrioid carcinoma	Survival
18	202111245	6604033001	35	2021.03.10	None	Negative	G2	Endometrioid carcinoma	Survival
19	202109376	6599082001	50	2021.03.02	None	Negative	G1	Endometrioid carcinoma	Survival
20	202108796	6597486001	50	2021.02.26	None	Negative	Unreported	Endometrioid carcinoma	Survival
21	202107617	6594099001	66	2021.02.22	None	Negative	G2	Endometrioid carcinoma	Survival
22	202107065	6593121001	54	2021.02.19	None	Negative	G2	Endometrioid carcinoma	Survival
23	202107040	6592659001	55	2021.02.19	None	Negative	G2	Endometrioid carcinoma	Survival
24	202106413	6591867002	62	2021.02.19	None	Negative	G3	Endometrioid carcinoma	Survival
25	202107061	6593127001	74	2021.02.18	None	Negative	Unreported	Endometrioid carcinoma	Survival
26	202106069	6591685001	48	2021.02.10	None	Negative	G2-G3	Endometrioid carcinoma	Survival
27	202106265	6592143001	67	2021.2.8	None	Negative	G1-G2	Endometrioid carcinoma	Survival
28	202105105	6589153001	66	2021.2.7	None	Negative	G2	Endometrioid carcinoma	Survival
29	202104610	6586632001	66	2021.1.29	None	Negative	G1-G2	Endometrioid carcinoma	Survival
30	202103335	6583614001	64	2021.1.22	have	Negative	G3	Endometrial plasma cancer	Survival
31	202102029	6580992001	41	2021.1.14	None	Negative	G2	Endometrioid carcinoma	Survival
32	202100916	6577821001	58	2021.1.7	None	Negative	G3	Endometrial plasma cancer	Survival
33	202100039	6575522001	46	2020.12.30	None	Negative	G1-G2	Endometrioid carcinoma	Survival
34	202100074	6574892001	53	2020.12.27	None	Negative	G1	Endometrioid carcinoma	Survival
35	202019859	6500305001	56	2020.07.01	None	Negative	G2-G3	Endometrioid carcinoma	Survival
36	202019651	6499426001	60	2020.06.30	None	Negative	G1-G2	Endometrioid carcinoma	Survival
37	202019012	6498498001	54	2020.06.24	None	Negative	G2	Endometrioid carcinoma	Survival
38	202018750	6498532001	55	2020.06.23	None	Negative	G2	Endometrioid carcinoma	Survival
39	202018535	6498394001	63	2020.06.22	None	Negative	G2-G3	Endometrioid carcinoma	Survival
40	202017597	6495559001	70	2020.06.16	None	Negative	G2	Endometrioid carcinoma	Survival
41	202016967	6494586001	67	2020.06.11	have	2	G3	Pellucidar carcinoma	Survival
42	202016954	6494553001	54	2020.06.11	None	Negative	G1	Endometrioid carcinoma	Survival
43	202016771	6492459001	51	2020.06.10	None	Negative	G2	Endometrioid carcinoma	Survival
44	202016519	6492559001	60	2020.06.09	None	Negative	G1-G2	Endometrioid carcinoma	Survival
45	202015981	6491878001	45	2020.06.08	None	Negative	G1	Endometrioid carcinoma	Survival
46	202016300	6490764001	58	2020.06.08	None	Negative	G1-G2	Endometrioid carcinoma	Survival
47	202015980	6492768001	35	2020.06.08	None	Negative	G1-G2	Endometrioid carcinoma	Survival
48	202016029	6491755001	80	2020.06.05	None	Negative	G2	Endometrioid carcinoma	Survival
49	202015175	6489123001	55	2020.06.01	None	Negative	G2	Endometrioid carcinoma	Survival
50	202015234	6490615001	62	2020.06.01	None	Negative	G2	Endometrioid carcinoma	Survival
51	202014955	6490007001	57	2020.05.29	have	1	G3	Endometrioid carcinoma	Survival
52	202014708	6489679001	47	2020.05.28	None	Negative	G1-G2	Endometrioid carcinoma	Survival
53	202014506	6488848001	62	2020.05.27	None	Negative	G2	Endometrioid carcinoma	Survival
54	202014520	6489152001	47	2020.05.27	None	Negative	G2	Endometrioid carcinoma	Survival
55	202014510	6487963001	49	2020.05.27	None	Negative	G1-G2	Endometrioid carcinoma	Survival
56	202014056	6485605001	80	2020.05.25	None	1	G3	Endometrial high-grade serous carcinoma	Survival
57	202013020	6485059001	44	2020.05.18	None	Negative	G1	Endometrioid carcinoma	Survival
58	202012722	6484019001	50	2020.05.15	None	Negative	G2	Endometrioid carcinoma	Survival
59	202012993	6484472001	60	2020.05.08	None	Negative	G2	Endometrioid carcinoma	Survival
60	202012751	6483862001	62	2020.05.07	None	Negative	G2	Endometrioid carcinoma	Survival
61	202013644	6481339001	52	2020.5.21	None	Negative	G1-G2	Endometrioid carcinoma	Survival
62	202156238	6715512	49	2021.11.19	None	None	None	Normal	Survival
63	202156757	6717028	50	2021.11.23	None	None	None	Normal	Survival
64	202156231	6715699	52	2021.11.19	None	None	None	Normal	Survival
65	202155673	6714427	49	2021.11.17	None	None	None	Normal	Survival
66	202149972	6698487	49	2021.10.14	None	None	None	Normal	Survival
67	202143163	6681280	48	2021.08.31	None	None	None	Normal	Survival
68	202143070	6680068	52	2021.08.31	None	None	None	Normal	Survival
69	202141854	6678997	47	2021.08.24	None	None	None	Normal	Survival
70	202142096	6679308	49	2021.08.25	None	None	None	Normal	Survival
71	202142075	6678599	50	2021.08.25	None	None	None	Normal	Survival
72	202136772	6664964	51	2021.07.28	None	None	None	Normal	Survival
73	202136774	6666585	48	2021.07.28	None	None	None	Normal	Survival
74	202138934	6671860	48	2021.08.09	None	None	None	Normal	Survival
75	202137305	6667547	76	2021.07.30	None	None	None	Normal	Survival
76	202117811	6619209	47	2021.04.16	None	None	None	Normal	Survival
77	202118111	6619915	52	2021.04.19	None	None	None	Normal	Survival
78	202119197	6623833	45	2021.04.23	None	None	None	Normal	Survival
79	202114596	6613130	49	2021.03.30	None	None	None	Normal	Survival
80	202111251	6604016	42	2021.03.11	None	None	None	Normal	Survival
81	202109890	6600495	52	2021.03.03	None	None	None	Normal	Survival
82	202109898	6600904	48	2021.03.04	None	None	None	Normal	Survival
83	202110145	6602294	47	2021.03.05	None	None	None	Normal	Survival
84	202109536	6600719	51	2021.03.03	None	None	None	Normal	Survival
85	202109905	6601589	47	2021.03.04	None	None	None	Normal	Survival
86	202109897	6601658	48	2021.03.03	None	None	None	Normal	Survival
87	202109033	6598672	52	2021.03.01	None	None	None	Normal	Survival
88	202109598	6601060	53	2021.03.03	None	None	None	Normal	Survival
89	202109617	6601118	44	2021.03.03	None	None	None	Normal	Survival
90	202109551	6599684	47	2021.03.03	None	None	None	Normal	Survival
91	202110146	6602020	47	2021.03.05	None	None	None	Normal	Survival
92	202015004	6479061001	43	2020.04.09	None	None	None	Normal	Survival
93	202011325	6479134001	47	2020.05.06	None	None	None	Normal	Survival
94	202011536	6479946001	50	2020.05.07	None	None	None	Normal	Survival
95	202011542	6482013001	42	2020.05.07	None	None	None	Normal	Survival
96	202011947	6480248001	48	2020.05.11	None	None	None	Normal	Survival
97	202011981	6482230002	52	2020.05.11	None	None	None	Normal	Survival
98	202012341	6479690001	53	2020.05.13	None	None	None	Normal	Survival
99	202012368	6483831001	48	2020.05.13	None	None	None	Normal	Survival
100	202012147	6483659001	54	2020.05.12	None	None	None	Normal	Survival
101	202012726	6484238001	54	2020.05.15	None	None	None	Normal	Survival
102	202013014	6485651002	55	2020.05.18	None	None	None	Normal	Survival
103	202013363	6486699001	48	2020.05.20	None	None	None	Normal	Survival
104	202013646	6487409002	45	2020.05.21	None	None	None	Normal	Survival
105	202014604	6485209001	49	2020.05.25	None	None	None	Normal	Survival
106	202016055	6492823001	69	2020.06.05	None	None	None	Normal	Survival
107	202016541	6493684001	47	2020.06.09	None	None	None	Normal	Survival
108	202016524	6493190001	48	2020.06.09	None	None	None	Normal	Survival
109	202017200	6495411001	52	2020.06.12	None	None	None	Normal	Survival
110	202016761	6494119001	52	2020.06.10	None	None	None	Normal	Survival
111	202016770	6493590001	52	2020.06.10	None	None	None	Normal	Survival
112	202017234	6495271001	47	2020.06.12	None	None	None	Normal	Survival
113	202017390	6495910001	51	2020.06.15	None	None	None	Normal	Survival
114	202017430	6495818001	50	2020.06.15	None	None	None	Normal	Survival
115	202018499	6497245001	51	2020.06.23	None	None	None	Normal	Survival
116	202018718	6498236001	51	2020.06.23	None	None	None	Normal	Survival
117	202018749	6498631001	52	2020.06.23	None	None	None	Normal	Survival
118	202018800	6498286001	50	2002.06.23	None	None	None	Normal	Survival
119	202019235	6501483001	49	2020.06.28	None	None	None	Normal	Survival
120	202019476	6501596001	45	2020.06.29	None	None	None	Normal	Survival
121	202019847	6501060001	51	2020.07.01	None	None	None	Normal	Survival
